# Work-type influences perceived livestock herding success in Australian Working Kelpies

**DOI:** 10.1186/s40575-018-0063-y

**Published:** 2018-08-13

**Authors:** Jonathan B. Early, Elizabeth A. Arnott, Lisa J. Mascord, Diane van Rooy, Paul D. McGreevy, Claire M. Wade

**Affiliations:** Faculty of Science, University of Sydney, Camperdown, NSW 2006 Australia

**Keywords:** Kelpie, Behaviour, Livestock, Working-type

## Abstract

**Background:**

Working dog handlers and breeders have very different behavioural requirements in the animals that they employ for managing livestock. The Australian Working Kelpie breed may be used in several working contexts, notably yards, paddocks and a combination of both. The working context influences the skillsets required and gives rise to three corresponding work-types: *Yard, Paddock* and *Utility* Kelpies. In particular, dogs used for working stock in the confines of yards and trucks interact with stock more forcefully than those mustering in larger areas (paddocks) where they can herd stock effectively from a greater distance. This article explores owner assessments of dog working quality and assessment of genomic similarity by multidimensional scaling, to ask whether it is sufficient for breeders to aim for a multipurpose breeding objective, or whether breeding only specialist lines maximises user satisfaction for yard and paddock work.

**Results:**

Reported owner perceptions of 298 dogs assessed with the Livestock Herding Dog assessment tool showed that dog handlers across all working types were very happy with their dogs’ level of general skills.

Compared with both *Yard* and *Utility* Kelpies, *Paddock* Kelpies had significantly lower trait scores for *force* (pressure applied by the dog to move livestock), willingness to *back* the stock (run along a sheep’s dorsum) and *bite* (frequency of using the mouth to grab or bite the livestock). Meanwhile, compared with both Paddock and *Utility* Kelpies, the *Yard* Kelpies had significantly higher scores for hyperactivity and excitability (both with and without stock) and impulsiveness without stock. As one would predict for all-rounders, *Utility* Kelpies had intermediate scores for all behaviours and working traits.

**Conclusions:**

Specialist characteristics were displayed by dogs in the *Yard* Kelpie and *Paddock* Kelpie groups. In particular, *Yard* Kelpies demonstrate higher excitability, willingness to *back* the stock, and a higher tendency to *bark* and *bite* the stock. Conversely, *Paddock* Kelpies rarely display these characteristics. *Utility* Kelpies, as the name suggests, are intermediate between the other two groups and display the characteristics of both. Genetic analysis suggests that the Yard, Utility and Paddock Kelpies are not distinguishable at a DNA level. In conclusion, at this time there is no suggestion of a breed split in the Australian Working Kelpie generated by selection for work type. A common breeding objective should enable dogs to be produced that fulfil all potential working requirements. This reinforces the importance of breeder skill in recognising the phenotypic potential of pups in order to place them in appropriate working contexts.

**Electronic supplementary material:**

The online version of this article (10.1186/s40575-018-0063-y) contains supplementary material, which is available to authorized users.

## Plain English summary

Dogs from the Australian Working Kelpie breed were categorised by their owners and handlers into different working type categories. Dogs from this breed may be used in several working contexts, notably moving stock in the close quarters of stock yards, through large fields and a combination of both. The working context influences the skills required by the dog and gives rise to three corresponding work-types: *Yard, Paddock* and *Utility* Kelpies. We compared the work-type and personality attributes of dogs that were declared by their owners to be one of the three working types. *Yard* Kelpies demonstrated higher excitability, willingness to *back* the stock, and a higher tendency to *bark* and *bite* the stock. Conversely, *Paddock* Kelpies rarely displayed these characteristics. *Utility* Kelpies, as the name suggests, were intermediate between the other two groups and displayed the characteristics of both. Genetic analysis suggests that the Yard, Utility and Paddock Kelpies are not distinguishable at a DNA level suggesting that there is no current genetic breed split that is related to the different working types.

## Background

The Farm Dog Project at the University of Sydney aims to better understand the phenotypic behavioural attributes (traits and manoeuvres) that characterise excellent livestock herding dogs. It is well understood that there is a major breed split between Australian Working Kelpies (AWK) and conformation-bred Australian Kelpies (AK) [1]. However, people outside of the working dog community are largely unaware of a further perceived split among the AWK. While some AWK breeders specialise in producing dogs with specialised attributes suited to paddock (extensive) or yard (intensive) stock work, others aim to produce dogs that can “do it all”.

### The Australian working kelpie

The Australian Working Kelpie (AWK) was developed in the late nineteenth Century from three pairs of “Working Collies” imported into Australia from Scotland [2]. All of the early pairs were black and tan or solid black with little or no white markings [2]. Two bitches from the early intermingling of these pairs had the call-name “Kelpie”. One of the first to be bred from was “Gleeson’s Kelpie”. This animal was bred with an all-black dog “Moss” and one female pup from the resultant litter “King’s Kelpie” displayed outstanding working ability in herding trials, although the metrics supporting this assessment are unavailable. She went on to found the Kelpie breed. Breed registrations are maintained by the Working Kelpie Council of Australia and the registry is “open” allowing unregistered animals with good working ability to be crossed into the breed. Despite being tough and relatively free from inherited disorders, the so-called working failure resulting in cull of livestock herding dogs, chiefly Kelpies, in Australia has been estimated at around 20% [3].

### Three dominant working types within the AWK

Working types of Kelpie are detailed in other work [3–5], but briefly:

*Paddock* Kelpies are used to gather (muster) animals from extensive open fields and ranges. These dogs are required to show great intelligence (sagacity), work independently from the human handler, calmly and effectively gathering the stock without unduly disturbing them. They typically start work facing the front of the stock, running around the periphery of the mob in an extensive *cast* and then using their behavioural characteristics of *eye* and *hold,* pressure the animals into a single group that they can move calmly towards the handler, who typically remains at the mob’s targeted destination (such as a gate).

*Yard* Kelpies work at close quarters to the livestock, pushing them through networks of yards for the purposes of transit (e.g. loading onto trucks), husbandry (e.g. for shearing or routine medication) or slaughter. This working type typically uses forceful measures to move the stock out of corners and through tight spaces (*force, bark, bite)* and they may move rapidly and efficiently around the yarding system by *backing* the animals (the action of a dog jumping up onto a sheep’s backs to assist in moving those sheep that are at the head of the mob). Yard dogs work under the direction of the handler and may work either at the front or the rear of the stock.

*Utility* Kelpies are general purpose livestock herding dogs. These animals are expected to *muster* (the traits demonstrated by the *Paddock* dogs) but are also expected to do move animals around the stock-yards or onto trucks.

Among these types, the *Paddock* and *Yard* dogs are regarded as specialised while the *Utility* dog is a generalist type.

## Aims

In the current study, we analyse owners’ reports of the individual phenotypes of their dogs that were categorised by their owners into one of three working types (*Paddock, Yard* and *Utility*). Dogs that were categorised across multiple types are recorded as *Utility* dogs. We then explore the major working behaviour requirements of these types and ask whether it is possible in a single breeding program to breed dogs that have the required expression of every working characteristic to work across the spectrum and, if so, the extent to which users’ expectations of the “working-quality” of the dogs has to be moderated for the working context. Understanding these requirements will refine relevant breeding objectives for the three major working types and provide resources to direct dog buyers to appropriate breeders. Better matching of clients and breeders is expected to result in better welfare outcomes and reduced wastage.

## Methods

The Livestock Working (Herding) Dog Assessment Form was designed to elicit data from livestock working dog handlers on the perceived quality of performance of their dogs according to 63 working and behavioural metrics, described in detail elsewhere [6]. Of the responses recorded as of May 19 2017, 298 participants’ dogs were described as being of the Kelpie breed. Among these, 35 were described as *Yard* dogs, 115 as *Paddock* dogs and 145 as *Utility* working dogs. Responses were recorded via a web based survey tool that enables participation from handlers Australia wide. Pedigree information on survey participants was not available.

For each trait (such as *eye*) and desirable manoeuvre (such as *cast*), the descriptive metrics from the assessment form were converted to numerical scores (Additional file 1: Table S1) and Glossary. For these scores, means and variances were estimated within each of the three dog working-types. Dog phenotype scores for each trait and manoeuvre were compared across work-types (*Paddock* versus *Yard*, *Paddock* versus *Utility*, *Yard* versus *Utility*) using a *Welch’s* t-test [7] with Welch-Satterthwaite degrees of freedom. Probabilities were determined from critical values of the Student’s t-distribution using the *t.test* function in Microsoft Excel. Significant Welch’s t-test scores were used to define group characteristic traits and behaviours. Pair-wise comparisons were re-assessed for significance after multiple test correction for the 63 comparisons.

Traits were regarded as unique to a work-type if the work-type obtained a trait score distribution that was statistically significantly different, at the 0.05 level, from the trait score distributions of the other two work types.

Venous blood samples were obtained from 22 dogs and the samples transferred to Whatman FTA (Flinders Technology Associates) cards for submission to the genotyping supplier. A further 42 dogs were sampled using Performagene saliva collection kits (DNA Genotek, Ontario Canada) and DNA was extracted following standard kit-issued protocol. Samples were collected with University of Sydney animal ethics committee’s approval (N00/10–2012/3/5837 and N00/10–2012/3/5928). Genotyping was conducted on the Illumina Canine High Density Genotyping array (172,939 markers) by Neogen/Geneseek Nebraska USA.

The genetic similarity between working-type groups was assessed through the application of clustering and multi-dimensional scaling of genotyping data for 19 dogs classified as *Paddock* dogs, 11 dogs classified as *Yard* dogs and 34 dogs classified as *Utility* dogs in the package “Plink” [8].

## Results

Trait means and standard errors are shown in Additional file 1: Table S2.

Compared with both *Yard* and *Utility* dogs, *Paddock* dogs had significantly lower trait scores for *force* (pressure applied by the dog in order to move livestock), willingness to *back* the stock and *bite* (*frequency* – assessed on a scale from never (score 1) to very frequently (score 5)).

Participants rated the quality of their ability in the manoeuvres and traits of *cast*, *gather*, *force*, *cover, head, hold, balance, break, back, initiative, anticipation, trainability* and *natural-ability* (extremely poor (score 1) – excellent (score 5)). Working groups were rated with a mean *force* scores of 3.64 ± 1.1, 3.99 ± 0.73 and 4.15 ± 0.89 for *Paddock, Utility* and *Yard* groups, respectively (Additional file 1: Table S2). *Paddock* group scores for *force* were significantly lower than those of the other two working groups. Fifty-eight percent of *Paddock* dogs still scored either “very good” or “excellent” (compared with 70% for *Utility* and 80% for *Yard* dogs). With respect to the dog’s willingness to *back* the stock: only 23% of *Paddock* dogs scored as either “very good” or “excellent” compared with 50% of *Utility* dogs and 71% of *Yard* dogs. It should be recognised that *“*excellent” *force* is not necessarily maximum force and is more likely to be highly appropriate force.

Compared with both *Paddock* and *Utility* dogs, the *Yard* dogs had significantly higher scores for *hyperactivity* and *excitability* (both with and without stock) and *impulsiveness* without stock (Table 1). They are also reported to take more time between stimulation (commands) and response (longer *latency* to respond). Their defining feature was a significantly higher mean score to *back* the stock. Unsurprisingly, as *bite* is a frequent requirement of the *Yard* work-type, *Yard* dogs were reported to *bite*/nip stock more frequently. The *Yard* dogs had significantly lower scores for *calmness* (with and without stock), less *patience* with stock, less ability to *cast, gather, head* or *hold* the stock than other working types. They also showed less *eye* (i.e., standing with their head lowered in a predatory stance, staring intently at the stock) and less *balance* when working stock (the ability of the dog to judge the optimal working distance from the livestock). They also attracted lower scores for *break* quality (the movement a dog performs to move around and redirect livestock, usually when some animals separate from the main group).

**Table 1:** Pairwise comparison of work-type in the Australian Working Kelpie over 63 traits

Comparison	Trait	Welch’s t-test (unequal size& unequal variance)^a^	Degrees of freedom (Welch-Satterthwaite)	Probability
Paddock versus Yard	confidence_stock	−0.641	59	0.3226
	calmness_stock	3.033	61	**0.0051** ^b^
	intelligence_stock	1.133	54	0.2080
	trainability_stock	0.915	58	0.2603
	boldness_stock	−0.923	55	0.2582
	patience_stock	2.864	73	**0.0078**
	timidness_stock	1.453	58	0.1383
	persistence_stock	0.346	55	0.3737
	hyperactivity_stock	−3.485	53	**0.0015**
	initiative_stock	0.565	60	0.3378
	excitability_stock	−4.267	58	**0.0001** ^*^
	obedience_stock	−0.096	48	0.3950
	nervousness_stock	−0.054	56	0.3965
	impulsiveness_stock	−3.269	57	**0.0027**
	stamina	−0.536	63	0.3436
	confidence_without_stock	−1.839	59	0.0746
	calmness_without_stock	2.082	49	**0.0477**
	intelligence_without_stock	0.135	57	0.3935
	trainability_without_stock	0.000	57	0.3972
	boldness_without_stock	−0.100	60	0.3952
	patience_without_stock	1.156	56	0.2027
	timidness_without_stock	0.607	57	0.3295
	persistence_without_stock	0.794	58	0.2887
	hyperactivity_without_stock	−2.955	58	**0.0063**
	initiative_without_stock	−1.154	68	0.2037
	excitability_without_stock	−2.401	56	**0.0244**
	obedience_without_stock	0.321	50	0.3766
	nervousness_without_stock	−0.397	62	0.3668
	impulsiveness_without_stock	−3.407	58	**0.0018**
	sociability	−0.047	54	0.3967
	friendliness	1.133	55	0.2081
	cast	2.896	51	**0.0076**
	gather	3.788	51	**0.0006** ^*^
	force	−2.692	59	**0.0123**
	cover	2.177	51	**0.0394**
	head	2.322	52	**0.0291**
	hold	1.842	50	0.0744
	balance	2.499	57	**0.0195**
	break	0.657	57	0.3191
	back	−5.864	61	**0.0000** ^*^
	initiative	−1.333	57	0.1631
	anticipation	−0.369	51	0.3704
	trainability	− 0.731	56	0.3028
	natural_ability	1.843	51	0.0742
	eye	1.178	49	0.1974
	confidence_level	−0.149	49	0.3924
	calmness_level	2.305	57	**0.0300**
	boldness	−1.624	47	0.1071
	bark	− 1.755	63	0.0862
	bite	−1.462	59	0.1367
	cast	0.701	42	0.3089
	force	−3.540	51	**0.0013**
	bite_frequency	−5.615	65	**0.0000** ^*^
	bark_frequency	−1.597	51	0.1116
	overall_ability	0.000	59	0.3973
	obedience_come	1.075	54	0.2217
	obedience_sit	0.239	49	0.3855
	obedience_stay	1.922	56	0.0643
	listening	−0.092	53	0.3953
	latency	−3.091	46	**0.0047**
	tricks	−1.211	43	0.1896
	distraction	−1.554	44	0.1191
	fetch	0.273	31	0.3807
Paddock versus Utility				
	confidence_stock	−1.921	198	0.0635
	calmness_stock	0.152	235	0.3939
	intelligence_stock	0.575	233	0.3377
	trainability_stock	−0.086	233	0.3970
	boldness_stock	−1.367	215	0.1564
	patience_stock	0.235	228	0.3876
	timidness_stock	0.489	243	0.3535
	persistence_stock	0.486	222	0.3539
	hyperactivity_stock	0.000	227	0.3985
	initiative_stock	−0.585	231	0.3356
	excitability_stock	−0.416	226	0.3654
	obedience_stock	−0.432	239	0.3630
	nervousness_stock	0.165	240	0.3931
	impulsiveness_stock	−1.337	251	0.1630
	stamina	−0.349	221	0.3749
	confidence_without_stock	−1.694	225	0.0951
	calmness_without_stock	−0.647	233	0.3230
	intelligence_without_stock	1.381	245	0.1535
	trainability_without_stock	−0.675	235	0.3170
	boldness_without_stock	−1.943	217	0.0608
	patience_without_stock	0.247	231	0.3865
	timidness_without_stock	0.950	226	0.2534
	persistence_without_stock	0.168	219	0.3929
	hyperactivity_without_stock	0.000	223	0.3985
	initiative_without_stock	−1.715	235	0.0919
	excitability_without_stock	−1.706	221	0.0933
	obedience_without_stock	−0.412	224	0.3660
	nervousness_without_stock	1.033	222	0.2336
	impulsiveness_without_stock	−1.134	234	0.2093
	sociability	−0.307	225	0.3800
	friendliness	−0.350	206	0.3747
	cast	− 1.336	223	0.1633
	gather	−0.089	225	0.3969
	force	−2.879	216	**0.0067**
	cover	−0.597	225	0.3332
	head	−0.623	231	0.3279
	hold	−1.910	228	0.0648
	balance	−1.104	228	0.2164
	break	−2.218	230	**0.0346**
	back	−4.696	231	**0.0000** ^*^
	initiative	−1.121	230	0.2124
	anticipation	−1.613	233	0.1087
	trainability	−0.808	226	0.2872
	natural_ability	−0.824	209	0.2834
	eye	0.325	250	0.3780
	confidence_level	−0.538	229	0.3446
	calmness_level	−0.503	220	0.3509
	boldness	−2.377	218	**0.0242**
	bark	−0.076	231	0.3973
	bite	−1.761	250	0.0848
	cast	−0.888	245	0.2685
	force	−1.392	222	0.1511
	bite_frequency	−2.379	235	**0.0240**
	bark_frequency	−2.070	240	**0.0473**
	overall_ability	−1.767	213	0.0839
	obedience_come	−0.480	237	0.3551
	obedience_sit	0.492	244	0.3530
	obedience_stay	0.315	240	0.3792
	listening	0.072	241	0.3975
	latency	−1.307	212	0.1695
	tricks	−0.080	210	0.3972
	distraction	−0.133	191	0.3949
	fetch	0.836	139	0.2803
Yard versus Utility				
	confidence_stock	−0.582	43	0.3338
	calmness_stock	−3.058	52	**0.0050**
	intelligence_stock	−0.806	47	0.2854
	trainability_stock	−1.016	50	0.2358
	boldness_stock	0.103	44	0.3945
	patience_stock	−2.877	58	**0.0078**
	timidness_stock	−1.144	54	0.2054
	persistence_stock	−0.052	45	0.3962
	hyperactivity_stock	3.641	46	**0.0010**
	initiative_stock	−1.010	51	0.2373
	excitability_stock	4.215	48	**0.0002** ^*^
	obedience_stock	−0.148	44	0.3923
	nervousness_stock	0.167	51	0.3913
	impulsiveness_stock	2.310	58	**0.0296**
	stamina	0.319	50	0.3769
	confidence_without_stock	0.766	49	0.2946
	calmness_without_stock	−2.520	44	**0.0192**
	intelligence_without_stock	0.828	54	0.2807
	trainability_without_stock	−0.462	50	0.3561
	boldness_without_stock	−1.233	47	0.1848
	patience_without_stock	−1.043	48	0.2291
	timidness_without_stock	0.000	48	0.3968
	persistence_without_stock	− 0.731	46	0.3024
	hyperactivity_without_stock	3.130	48	**0.0042**
	initiative_without_stock	−0.128	58	0.3939
	excitability_without_stock	1.415	46	0.1458
	obedience_without_stock	−0.573	43	0.3354
	nervousness_without_stock	1.166	50	0.2003
	impulsiveness_without_stock	2.770	50	**0.0104**
	sociability	−0.146	46	0.3924
	friendliness	−1.435	43	0.1417
	cast	−3.819	43	**0.0006** ^*^
	gather	−4.002	44	**0.0004** ^*^
	force	0.904	47	0.2624
	cover	−2.621	44	**0.0152**
	head	−2.791	45	**0.0101**
	hold	−3.015	43	**0.0058**
	balance	−3.363	48	**0.0022**
	break	−2.177	48	**0.0395**
	back	2.780	52	**0.0101**
	initiative	0.634	49	0.3235
	anticipation	−0.600	45	0.3302
	trainability	0.236	47	0.3856
	natural_ability	−2.422	41	0.0239
	eye	−0.986	49	0.2428
	confidence_level	−0.154	43	0.3918
	calmness_level	−2.776	46	**0.0104**
	boldness	0.434	40	0.3600
	bark	1.791	53	0.0812
	bite	0.176	59	0.3911
	cast	−1.131	41	0.2081
	force	2.881	43	**0.0082**
	bite_frequency	4.084	55	**0.0002** ^*^
	bark_frequency	0.372	47	0.3697
	overall_ability	−1.209	46	0.1902
	obedience_come	−1.418	48	0.1452
	obedience_sit	0.048	46	0.3963
	obedience_stay	−1.759	51	0.0858
	listening	0.140	51	0.3930
	latency	2.434	41	**0.0233**
	tricks	1.199	40	0.1921
	distraction	1.519	40	0.1255
	fetch	0.235	26	0.3838

^*^Comparisons remaining significant after multiple-test correction for 63 tests (*p* < 0.000794)

^a^Negative scores indicate a lower trait mean score for the first listed group (e.g. Paddock in Paddock versus Yard)

^b^Probabilities < 0.05 are highlighted

*Utility* dogs had scores that were intermediate between the *Yard* and *Paddock* dogs for *bite* and *back*. Indeed, all three groups differed significantly for these two traits. In general, the behaviour scores of the *Utility* work-type clustered more closely with the *Paddock* work-type than the Yard work-type. Of the 63 traits and manoeuvres measured, only six differed significantly between *Paddock* and *Utility* dogs (*force, break, back, boldness*, *bite-frequency* and *bark-frequency*). Between *Yard* and *Utility* dogs, 21 characteristics differed significantly. Between *Yard* and *Paddock* dogs, 20 characteristics differed (18 of which were the same as those differing between the *Yard* and *Utility* dogs).

*Overall-ability*, *natural-ability* and *trainability* did not differ significantly among working-type groups. *Overall-ability* (scored between “worst dog I have ever seen/trained” to “best dog I have ever seen/trained) is perceived by breeders and handlers to represent a culmination of breeding, training and handling”. *Natural-ability* is regarded as the dog’s inherent talent for the working tasks and is thought to more likely represent genetic potential. *Trainability* is the ease with which the dog can be trained to accomplish the skills required for its working context.

**Fig. 1:**
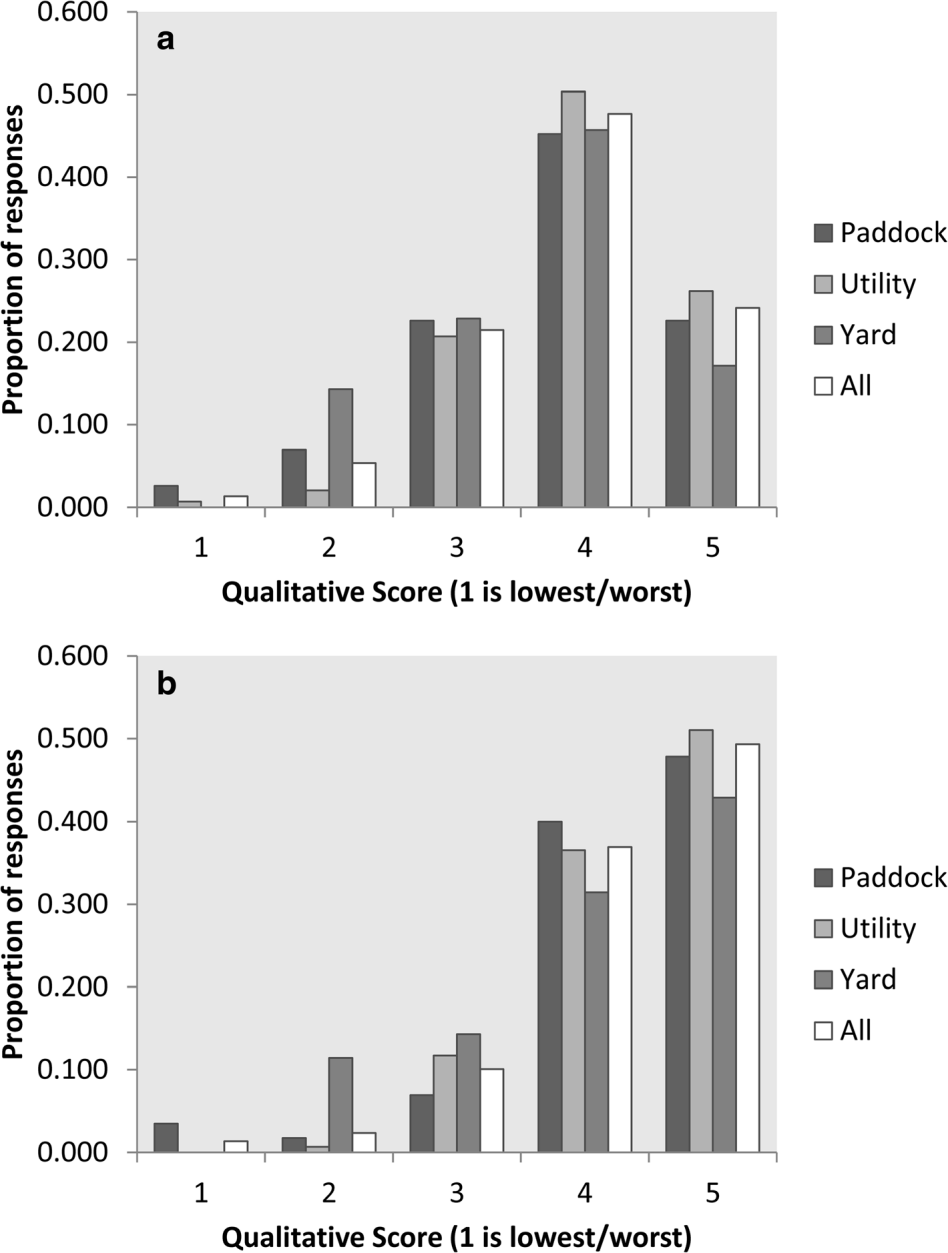
Proportion of dogs at each qualitative trait score (1 = worst, 5 = best) for **a** *overall-ability* and **b** *natural-ability* by work-type in the Australian Working Kelpie

The proportions of dogs at each scoring level for the traits of *Overall-ability* and *Natural-ability* across working types are shown in Fig. 1a and b respectively.

**Fig. 2:**
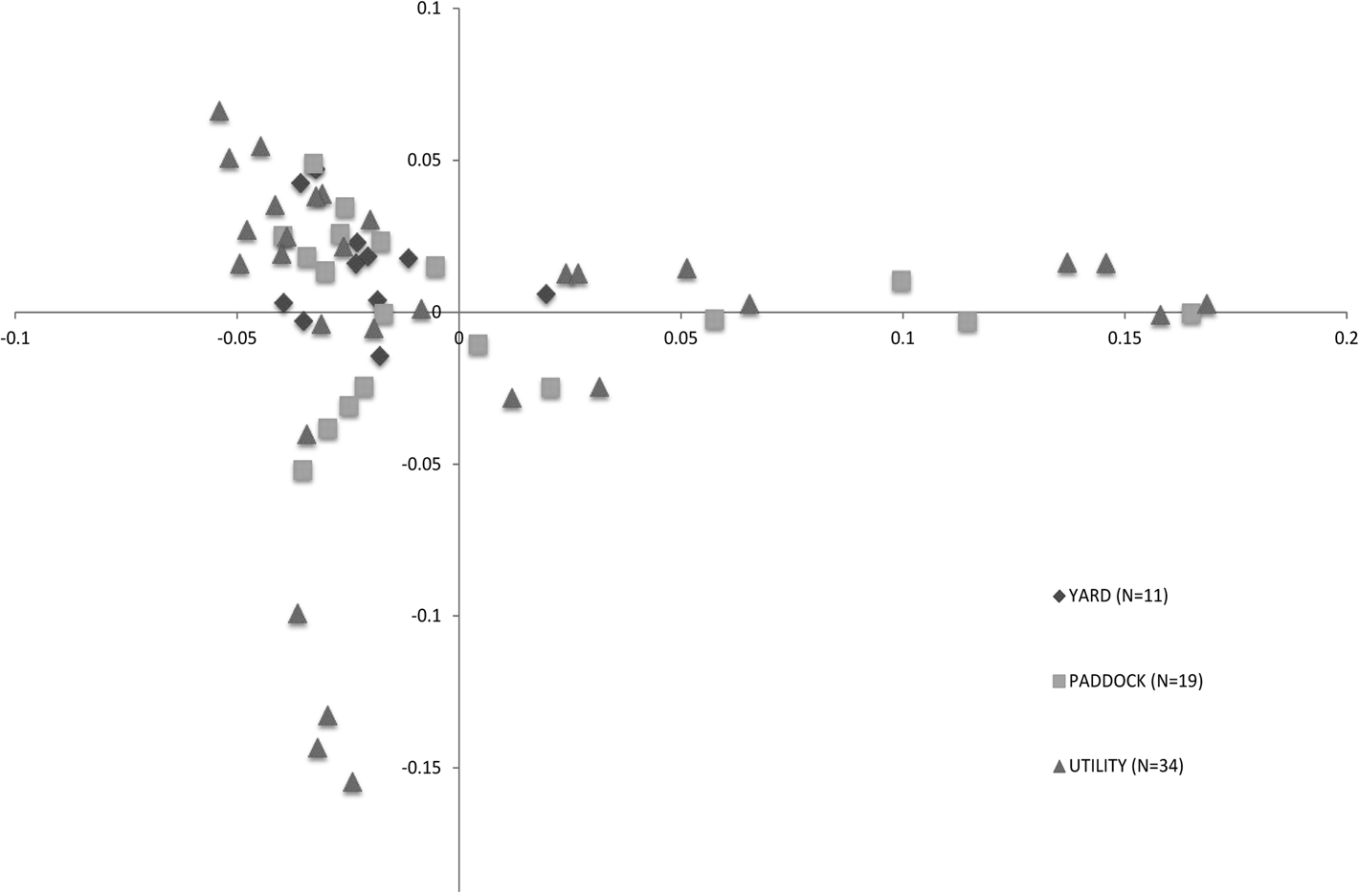
Multi-dimensional scaling plot displaying genetic distances between individuals from Australian Working Kelpie populations described as *Paddock*, *Yard* and *Utility* working types demonstrating that there is no clear genetic differentiation between the working types (Table S3). (Legend: Paddock (*N* = 19) –square marker; Yard (*N* = 11) – diamond marker; Utility (*N* = 34) – triangular marker)

*Paddock* dogs, *Yard* dogs and *Utility* dogs were unable to be genetically differentiated on a whole-genome level (that is, the genomic inflation estimate of lambda (based on median chi-squared statistic = 1). Similarly, clustering analysis identified dogs in the analysis as a single genetic cluster although there is some evidence of potential cross-breeding within the study population as is evidenced by the directional trends in the data for the *Paddock* and *Utility* dogs (Fig. 2). Genetically, *Yard* dogs were centrally located in the Australian Working Kelpie population cluster based on genetic variation.

## Discussion

This study of herding dog owners’ reports of their dogs’ behavioural attributes clearly demonstrates that, over the three work-types of dogs assessed, owners and handlers held their dogs in high regard. For example, the majority of participant dogs (86%) were assessed as having *overall-ability* that was “above average” or “the best dog I have ever owned/trained” and had “good” or “excellent” *natural-ability*. Conversely, very few dogs rated poorly for their *overall-abili*ty; with only 6% being judged as “below average” or “one of the worst dogs I have ever owned/trained”. Even fewer (~ 4%) were judged to have “extremely poor” or “poor” *natural-ability*. Of course, this may reflect respondent bias in that owners of dogs that are currently disappointing may be disinclined to spend time describing them for research purposes or it is possible that dogs already assessed as poor are no longer with them. Compared with the owners of *Paddock* and *Utility* dogs, the owners of *Yard* dogs were more likely to be critical of their dogs; with 11% being rated as “below average” or “one of the worst dogs I have ever owned/trained” and only 74% rated at “above average” or “one of the best dogs I have ever owned/trained” (Fig. 1). The relatively small number of *Yard* dogs assessed means that it is possible that these ratings reflect a form of sampling error.

Two traits (*bite* and *back*) differed significantly across all three groups and these, along with *force,* uniquely differentiated the *Paddock* dogs (which had the lowest scores for all three attributes). *Yard* dogs had significant strengths in several attributes pertaining to energy level, vocalisation and intensity of interaction with stock (*calmness, hyperactivity, excitability, bark, bite, back,* and *patience*). In contrast, they also had significantly lower scores for the trained manoeuvres of particular value in the context of paddock. It is possible that this finding is a function of training and exposure, rather than innate talent. Across all of the assessed attributes, *Yard* dogs were the most differentiated group but only 35 of 298 dogs were used for this purpose. The higher level of bark and bite demonstrated by the yard dogs is a characteristic of the desirability of these traits in the work context.

This work underlies a broader project goal to create a breeding program aiming to reduce loss of dogs from the industry through their being unsuited to the purpose for which they were bought. Our work demonstrates that separate breeding objectives for the groups are not required. The three work-types of dogs partitioned in this analysis did not differ significantly in *overall-ability*, *natural-ability* or *trainability*, suggesting that breeding for “all-rounders” does not endanger the global working quality of this breed when dogs are used in their correct context. This indicative finding was also supported by the DNA analysis that showed that the work-types did not cluster separately at the genetic level. Despite this, people employing different working types have very different perceptions of what attributes are acceptable and desirable. For any breeding program that aims to influence the prevalence of a range of attributes, there will always be a distribution of quality for individual characters produced in any kennel.

Given the relatively limited demand for *Yard* dogs, it is expected that most breeders would rather specialise in either *Paddock* dogs or *Utility* dogs and then on occasion be able to effectively identify the outlier pups (from *Utility* and *Paddock* lines) with especially strong *Yard* attributes.

Mapping genes for *bite* and *back* which are the attributes that critically qualify the dogs for purpose might be central to the early identification of working homes for dogs, particularly for animals bred in *Utility* kennels. Alternatively, identifying other early predictors of these traits via behavioural testing would enhance welfare outcomes.

## Conclusions

Specialist characteristics were displayed by dogs in the *Yard* Kelpie and *Paddock* Kelpie groups. In particular, *Yard* Kelpies demonstrate higher excitability, willingness to *back* the stock, and a higher tendency to *bark* and *bite* the stock. Conversely, *Paddock* Kelpies rarely display these characteristics. *Utility* Kelpies, as the name suggests, are intermediate between the other two groups and display the characteristics of both. Genetic analysis suggests that the Yard, Utility and Paddock Kelpies are not distinguishable at a DNA level. In conclusion, at this time there is no suggestion of a breed split in the Australian Working Kelpie generated by selection for work type. A common breeding objective should enable dogs to be produced that fulfil all potential working requirements. This reinforces the importance of breeder skill in recognising the phenotypic potential of pups in order to place them in appropriate working contexts.

## Additional file

Additional file 1: Supplementary methods and tables. (DOCX 44 kb)

## Glossary

Backing: action of a dog jumping up onto sheep’s backs in order to assist in moving them in tight spaces such as in yards, sheds or trucks.
Balance: position a dog assumes in relation to the livestock and the handler that is best suited to move the livestock to the desired location efficiently.
Break: Type of movement a dog performs to move around and redirect livestock usually when some animals separate from the main group.
Cast: initial movement of a dog around to the far side, in relation to the handler, of the livestock in order to gather and deliver them back towards the handler.
Cover: type of movement a dog uses around livestock while keeping them together.
Eye: postural behaviour that involves staring at livestock from a stationary position or involve stalking-like movement. Considered to be a remnant of stalking behaviour that forms part of the predatory sequence in wild dogs and wolves.
Force: pressure applied by the dog in order to move livestock.
Heading: movement of a dog to the front of a group of livestock to stop or redirect their movement.
Hold: the action of a dog to keep livestock together.: From: McGreevy et al. [6]. Barton (ACT), Australia: Rural Industries Research and Development Corporation.
