# Supplementary material for: Work-type influences perceived livestock herding success in Australian Working Kelpies
Source: Canine Genet Epidemiol. 2018 Aug 13;5:5. doi: 10.1186/s40575-018-0063-y (PMC6090621; doi:10.1186/s40575-018-0063-y)
Supplement: Supplementary file 1 — Supplementary methods and tables. (DOCX 44 kb) [file 40575_2018_63_MOESM1_ESM.docx]

Supplementary Materials

Scoring of data

Individual working phenotypes (Table S 1) were scored on either a 5 or a 6 point Likert scale and given a qualitative score of 1 to 5 or 1 to 6 for least desirable to most desirable categories (that are described as footnotes a., b. , c., d., f. and g. in Table S1). The exceptions were traits scored for appropriateness, where a score of 3 was optimal (i.e. those with descriptive range described with footnote e. in Table S1). Traits with a not-observed/not-applicable option included a score of -9 which was ignored in the analysis.

Individual working phenotypes (n=63) were compared pairwise between working-types using a Welch’s *t*-test for unequal means and unequal variances[[1](#_ENREF_1)]. Degrees of freedom were calculated using the Welch-Satterthwaite equation. Calculations were performed in Microsoft Excel with equations entered manually into cells after calculating mean and sample variance for each trait within working type.

1. Welch, B.L., *The generalization of "student's" problem when several different population variances are involved.* Biometrika, 1947. **34**(1-2): p. 28-35.

Table S1 Individually scored traits, descriptive scales, numeric scales

| Trait ID | Trait | Question Section | Paddock | Yard | Utility | Total | Scoring scale | Descriptive range |
| --- | --- | --- | --- | --- | --- | --- | --- | --- |
| 1 | confidence | A. | 114 | 34 | 142 | 290 | 1 to 5,-9 | a. |
| 2 | calmness | A. | 114 | 34 | 142 | 290 | 1 to 5,-9 | a. |
| 3 | intelligence | A. | 114 | 34 | 142 | 290 | 1 to 5,-9 | a. |
| 4 | trainability | A. | 114 | 34 | 142 | 290 | 1 to 5,-9 | a. |
| 5 | boldness | A. | 114 | 34 | 142 | 290 | 1 to 5,-9 | a. |
| 6 | patience | A. | 114 | 34 | 142 | 290 | 1 to 5,-9 | a. |
| 7 | timidness | A. | 114 | 34 | 142 | 290 | 1 to 5,-9 | a. |
| 8 | persistence | A. | 114 | 34 | 142 | 290 | 1 to 5,-9 | a. |
| 9 | hyperactivity | A. | 114 | 34 | 142 | 290 | 1 to 5,-9 | a. |
| 10 | initiative taking | A. | 114 | 34 | 142 | 290 | 1 to 5,-9 | a. |
| 11 | excitability | A. | 114 | 34 | 142 | 290 | 1 to 5,-9 | a. |
| 12 | obedience | A. | 114 | 34 | 142 | 290 | 1 to 5,-9 | a. |
| 13 | nervousness | A. | 114 | 34 | 142 | 290 | 1 to 5,-9 | a. |
| 14 | impulsiveness | A. | 114 | 34 | 142 | 290 | 1 to 5,-9 | a. |
| 15 | stamina | A. | 114 | 34 | 142 | 290 | 1 to 5,-9 | a. |
| 16 | confidence | B. | 114 | 34 | 142 | 290 | 1 to 5,-9 | a. |
| 17 | calmness | B. | 114 | 34 | 142 | 290 | 1 to 5,-9 | a. |
| 18 | intelligence | B. | 114 | 34 | 142 | 290 | 1 to 5,-9 | a. |
| 19 | trainability | B. | 114 | 34 | 142 | 290 | 1 to 5,-9 | a. |
| 20 | boldness | B. | 114 | 34 | 142 | 290 | 1 to 5,-9 | a. |
| 21 | patience | B. | 114 | 34 | 142 | 290 | 1 to 5,-9 | a. |
| 22 | timidness | B. | 114 | 34 | 142 | 290 | 1 to 5,-9 | a. |
| 23 | persistence | B. | 114 | 34 | 142 | 290 | 1 to 5,-9 | a. |
| 24 | hyperactivity | B. | 114 | 34 | 142 | 290 | 1 to 5,-9 | a. |
| 25 | initiative | B. | 114 | 34 | 142 | 290 | 1 to 5,-9 | a. |
| 26 | excitability | B. | 114 | 34 | 142 | 290 | 1 to 5,-9 | a. |
| 27 | obedience | B. | 114 | 34 | 142 | 290 | 1 to 5,-9 | a. |
| 28 | nervousness | B. | 114 | 34 | 142 | 290 | 1 to 5,-9 | a. |
| 29 | impulsiveness (has sudden, strong urges to act; acts without forethought; acts without considering effects of actions | B. | 114 | 34 | 142 | 290 | 1 to 5,-9 | a. |
| 30 | sociability | B. | 114 | 34 | 142 | 290 | 1 to 5,-9 | a. |
| 31 | friendliness | B. | 114 | 34 | 142 | 290 | 1 to 5,-9 | a. |
| 32 | cast | C. | 114 | 34 | 142 | 290 | 1 to 5 | b. |
| 33 | gather | C. | 114 | 34 | 142 | 290 | 1 to 5 | b. |
| 34 | force | C. | 114 | 34 | 142 | 290 | 1 to 5 | b. |
| 35 | cover | C. | 114 | 34 | 142 | 290 | 1 to 5 | b. |
| 36 | head | C. | 114 | 34 | 142 | 290 | 1 to 5 | b. |
| 37 | hold | C. | 114 | 34 | 142 | 290 | 1 to 5 | b. |
| 38 | balance | C. | 114 | 34 | 142 | 290 | 1 to 5 | b. |
| 39 | break | C. | 114 | 34 | 142 | 290 | 1 to 5 | b. |
| 40 | back | C. | 114 | 34 | 142 | 290 | 1 to 5 | b. |
| 41 | initiative | C. | 114 | 34 | 142 | 290 | 1 to 5 | b. |
| 42 | anticipation | C. | 114 | 34 | 142 | 290 | 1 to 5 | b. |
| 43 | trainability | C. | 114 | 34 | 142 | 290 | 1 to 5 | b. |
| 44 | Natural ability | C. | 114 | 34 | 142 | 290 | 1 to 5 | b. |
| 45 | eye | C. | 114 | 34 | 142 | 290 | 1 to 6 | c. |
| 46 | confidence | C. | 114 | 34 | 142 | 290 | 1 to 5,-9 | a. |
| 47 | calmness | C. | 114 | 34 | 142 | 290 | 1 to 5,-9 | a. |
| 48 | boldness | C. | 114 | 34 | 142 | 290 | 1 to 5 | d. |
| 49 | bark appropriateness | C. | 114 | 34 | 142 | 290 | 1,3,5 | e. |
| 50 | bite appropriateness | C. | 114 | 34 | 142 | 290 | 1,3,5 | e. |
| 51 | cast appropriateness | C. | 114 | 34 | 142 | 290 | 1,3,5 | e. |
| 52 | force appropriateness | C. | 114 | 34 | 142 | 290 | 1,3,5 | e. |
| 53 | bite frequency | C. | 114 | 34 | 142 | 290 | 1 to 6 | f. |
| 54 | bark frequency | C. | 114 | 34 | 142 | 290 | 1 to 6 | f. |
| 55 | Overall ability | C. | 114 | 34 | 142 | 290 | 1 to 5 | g. |
| 56 | obedience come | D. | 114 | 34 | 142 | 290 | 1 to 5, -9 | f. |
| 57 | obedience sit | D. | 114 | 34 | 142 | 290 | 1 to 5, -9 | f. |
| 58 | obedience stay | D. | 113 | 34 | 142 | 289 | 1 to 5, -9 | f. |
| 59 | listening | D. | 111 | 34 | 139 | 284 | 1 to 5, -9 | f. |
| 60 | latency | D. | 103 | 32 | 133 | 268 | 1 to 5, -9 | f. |
| 61 | tricks | D. | 102 | 32 | 130 | 264 | 1 to 5, -9 | f. |
| 62 | distraction | D. | 91 | 30 | 119 | 240 | 1 to 5, -9 | f. |
| 63 | fetch | D. | 71 | 20 | 93 | 184 | 1 to 5, -9 | f. |

A. Please rate how the dog is when working with stock. For each of the traits, please tick one of the five boxes (from ‘Very low’ to ‘Very high’) to best describe this dog.

B. Please rate how the dog is in situations without/away from stock.

C. Please rate how and how well the dog works with stock.

D. Some dogs are more obedient and trainable than others. By marking the appropriate choices, please indicate how trainable or obedient your dog has been in each of the following situations in the recent past.

a.     very low, low, average, high, very high, [not observed, not applicable]

b.    extremely poor, poor, average, good, excellent, [not observed, not applicable]

c.     none, weak, light, medium, strong, over

d.    extremely shy, shy, moderate, bold, extremely bold

e.    inadequate, appropriate, excessive

f.     never, seldom, sometimes, usually, always, [not observed/not applicable]

g.     one of the worst dogs I have ever seen/trained, below average, about average, one of the best dogs I have ever seen/trained

Table S2: Means and standard errors for each characteristic by working-type

| Trait | Paddock Mean | Paddock standard error | Utility Mean | Utility standard error | Yard Mean | Yard standard error |
| --- | --- | --- | --- | --- | --- | --- |
| confidence_stock | 4.00 | 1.06 | 4.22 | 0.54 | 4.12 | 0.87 |
| calmness_stock | 3.73 | 1.16 | 3.71 | 1.01 | 3.15 | 0.90 |
| intelligence_stock | 4.28 | 0.74 | 4.22 | 0.63 | 4.09 | 0.73 |
| trainability_stock | 3.92 | 0.91 | 3.93 | 0.77 | 3.76 | 0.77 |
| boldness_stock | 3.69 | 1.16 | 3.86 | 0.75 | 3.88 | 1.10 |
| patience_stock | 3.57 | 1.13 | 3.54 | 0.90 | 3.09 | 0.62 |
| timidness_stock | 2.11 | 0.94 | 2.05 | 0.97 | 1.85 | 0.81 |
| persistence_stock | 3.98 | 1.10 | 3.92 | 0.79 | 3.91 | 1.07 |
| hyperactivity_stock | 2.71 | 1.73 | 2.71 | 1.35 | 3.62 | 1.80 |
| initiative_stock | 3.84 | 0.98 | 3.91 | 0.81 | 3.74 | 0.77 |
| excitability_stock | 3.02 | 1.47 | 3.08 | 1.13 | 3.97 | 1.25 |
| obedience_stock | 3.80 | 0.87 | 3.85 | 0.82 | 3.82 | 1.21 |
| nervousness_stock | 1.96 | 0.94 | 1.94 | 0.90 | 1.97 | 0.88 |
| impulsiveness_stock | 2.32 | 1.15 | 2.51 | 1.44 | 2.97 | 1.00 |
| stamina | 4.00 | 0.96 | 4.04 | 0.68 | 4.09 | 0.67 |
| confidence_without_stock | 3.88 | 0.99 | 4.08 | 0.75 | 4.21 | 0.80 |
| calmness_without_stock | 3.84 | 1.04 | 3.92 | 0.88 | 3.38 | 1.35 |
| intelligence_without_stock | 4.31 | 0.63 | 4.17 | 0.67 | 4.29 | 0.55 |
| trainability_without_stock | 3.97 | 0.94 | 4.05 | 0.82 | 3.97 | 0.82 |
| boldness_without_stock | 3.48 | 1.22 | 3.73 | 0.83 | 3.50 | 0.98 |
| patience_without_stock | 3.69 | 1.02 | 3.66 | 0.84 | 3.47 | 0.93 |
| timidness_without_stock | 2.22 | 1.32 | 2.09 | 1.01 | 2.09 | 1.17 |
| persistence_without_stock | 3.74 | 1.04 | 3.72 | 0.72 | 3.59 | 0.90 |
| hyperactivity_without_stock | 2.65 | 1.82 | 2.65 | 1.32 | 3.38 | 1.53 |
| initiative_without_stock | 3.49 | 1.00 | 3.70 | 0.88 | 3.68 | 0.62 |
| excitability_without_stock | 2.77 | 1.82 | 3.04 | 1.30 | 3.38 | 1.65 |
| obedience_without_stock | 3.92 | 1.06 | 3.97 | 0.78 | 3.85 | 1.30 |
| nervousness_without_stock | 2.10 | 1.33 | 1.96 | 0.95 | 2.18 | 0.98 |
| impulsiveness_without_stock | 2.32 | 1.52 | 2.49 | 1.30 | 3.09 | 1.28 |
| sociability | 3.99 | 1.20 | 4.03 | 0.91 | 4.00 | 1.21 |
| friendliness | 4.31 | 1.02 | 4.35 | 0.58 | 4.09 | 0.98 |
| cast | 3.77 | 1.17 | 3.94 | 0.85 | 3.12 | 1.36 |
| gather | 3.99 | 0.90 | 4.00 | 0.68 | 3.24 | 1.06 |
| force | 3.64 | 1.10 | 3.99 | 0.73 | 4.15 | 0.89 |
| cover | 3.98 | 0.98 | 4.05 | 0.74 | 3.53 | 1.16 |
| head | 4.03 | 0.86 | 4.10 | 0.72 | 3.59 | 0.96 |
| hold | 3.82 | 0.92 | 4.04 | 0.73 | 3.44 | 1.17 |
| balance | 3.96 | 0.97 | 4.09 | 0.76 | 3.50 | 0.86 |
| break | 3.68 | 0.95 | 3.94 | 0.77 | 3.56 | 0.85 |
| back | 2.81 | 1.40 | 3.48 | 1.15 | 4.03 | 1.05 |
| initiative | 3.81 | 0.79 | 3.93 | 0.64 | 4.03 | 0.69 |
| anticipation | 3.93 | 0.84 | 4.11 | 0.72 | 4.00 | 0.97 |
| trainability | 3.90 | 0.88 | 3.99 | 0.67 | 4.03 | 0.81 |
| natural_ability | 4.25 | 0.92 | 4.34 | 0.55 | 3.88 | 1.10 |
| eye | 4.06 | 0.88 | 4.02 | 1.05 | 3.82 | 1.15 |
| confidence3 | 3.82 | 0.86 | 3.88 | 0.69 | 3.85 | 1.12 |
| calmness3 | 3.65 | 1.04 | 3.71 | 0.73 | 3.21 | 0.93 |
| boldness | 3.39 | 0.88 | 3.65 | 0.60 | 3.74 | 1.32 |
| bark | 2.61 | 1.17 | 2.62 | 0.97 | 2.94 | 0.85 |
| bite | 2.63 | 0.90 | 2.85 | 1.10 | 2.88 | 0.73 |
| cast | 2.80 | 0.63 | 2.89 | 0.67 | 2.65 | 1.37 |
| force | 2.73 | 1.07 | 2.90 | 0.78 | 3.50 | 1.29 |
| bite_frequency | 2.87 | 1.98 | 3.28 | 1.75 | 4.21 | 1.34 |
| bark_frequency | 2.85 | 1.74 | 3.19 | 1.66 | 3.29 | 2.06 |
| overall_ability | 3.82 | 0.97 | 4.02 | 0.61 | 3.82 | 0.78 |
| obedience_come | 4.39 | 0.72 | 4.44 | 0.65 | 4.21 | 0.74 |
| obedience_sit | 4.14 | 0.92 | 4.08 | 0.97 | 4.09 | 1.22 |
| obedience_stay | 3.99 | 1.02 | 3.95 | 1.02 | 3.62 | 0.95 |
| listening | 3.95 | 1.15 | 3.94 | 1.27 | 3.97 | 1.25 |
| latency | 2.21 | 1.17 | 2.39 | 1.02 | 2.97 | 1.57 |
| tricks | 2.05 | 0.93 | 2.06 | 0.82 | 2.34 | 1.54 |
| distraction | 2.27 | 1.20 | 2.29 | 1.14 | 2.67 | 1.59 |
| fetch | 2.21 | 2.57 | 2.01 | 1.95 | 2.10 | 2.52 |

Table S3 Data used in generating Figure 2.

| Breed_WorkType (Y= Yard, P=Paddock, U=Utility) | Identifier | C1 | C2 |
| --- | --- | --- | --- |
| Kelpie_Y | 1501 | -0.02325 | 0.016063 |
| Kelpie_Y | 1515 | -0.02295 | 0.022881 |
| Kelpie_Y | 1516 | -0.03503 | -0.00286 |
| Kelpie_Y | 1517 | -0.01836 | 0.004045 |
| Kelpie_Y | 1522 | -0.02053 | 0.018289 |
| Kelpie_Y | 1525 | -0.03568 | 0.042464 |
| Kelpie_Y | 1532 | -0.01137 | 0.017722 |
| Kelpie_Y | 1533 | -0.0323 | 0.047044 |
| Kelpie_Y | 1534 | 0.019622 | 0.006103 |
| Kelpie_Y | 1535 | -0.03955 | 0.003179 |
| Kelpie_Y | 1562 | -0.01786 | -0.01436 |
| Kelpie_P | 1473 | -0.03519 | -0.0519 |
| Kelpie_P | 1477 | -0.02957 | -0.03828 |
| Kelpie_P | 1510 | -0.0248 | -0.03085 |
| Kelpie_P | 1512 | -0.00529 | 0.015075 |
| Kelpie_P | 1513 | -0.02565 | 0.034524 |
| Kelpie_P | 1514 | -0.02678 | 0.025782 |
| Kelpie_P | 1521 | -0.01759 | 0.023239 |
| Kelpie_P | 1526 | -0.03274 | 0.048799 |
| Kelpie_P | 1528 | -0.03961 | 0.025061 |
| Kelpie_P | 1531 | -0.03014 | 0.013431 |
| Kelpie_P | 1658 | -0.02136 | -0.02449 |
| Kelpie_P | 1847 | 0.165026 | -0.00035 |
| Kelpie_P | 1848 | 0.004317 | -0.01056 |
| Kelpie_P | 1849 | 0.099667 | 0.010452 |
| Kelpie_P | 1850 | 0.114565 | -0.00295 |
| Kelpie_P | 1863 | 0.020643 | -0.02482 |
| Kelpie_P | 1880 | 0.057601 | -0.00233 |
| Kelpie_P | 1894 | -0.01693 | -0.00045 |
| Kelpie_P | 1895 | -0.03427 | 0.018142 |
| Kelpie_U | 1471 | -0.03428 | -0.0403 |
| Kelpie_U | 1472 | -0.00849 | 0.001094 |
| Kelpie_U | 1475 | -0.01918 | -0.00527 |
| Kelpie_U | 1496 | 0.051353 | 0.014452 |
| Kelpie_U | 1497 | -0.02006 | 0.030402 |
| Kelpie_U | 1498 | 0.137012 | 0.016237 |
| Kelpie_U | 1499 | 0.145767 | 0.016089 |
| Kelpie_U | 1500 | -0.03878 | 0.024814 |
| Kelpie_U | 1502 | -0.04142 | 0.035219 |
| Kelpie_U | 1503 | 0.031648 | -0.02468 |
| Kelpie_U | 1504 | -0.03639 | -0.09922 |
| Kelpie_U | 1505 | -0.02958 | -0.13269 |
| Kelpie_U | 1506 | -0.024 | -0.15464 |
| Kelpie_U | 1507 | -0.03189 | -0.1434 |
| Kelpie_U | 1508 | -0.03106 | -0.00389 |
| Kelpie_U | 1511 | -0.04781 | 0.027043 |
| Kelpie_U | 1519 | -0.0518 | 0.050736 |
| Kelpie_U | 1523 | -0.04472 | 0.054604 |
| Kelpie_U | 1524 | -0.03082 | 0.03886 |
| Kelpie_U | 1527 | -0.02603 | 0.021519 |
| Kelpie_U | 1529 | -0.05403 | 0.066263 |
| Kelpie_U | 1530 | -0.03225 | 0.038103 |
| Kelpie_U | 1561 | 0.024065 | 0.012702 |
| Kelpie_U | 1564 | 0.026799 | 0.012715 |
| Kelpie_U | 1657 | -0.03993 | 0.019079 |
| Kelpie_U | 1843 | 0.168472 | 0.002776 |
| Kelpie_U | 1844 | 0.158012 | -0.00089 |
| Kelpie_U | 1845 | 0.065472 | 0.002795 |
| Kelpie_U | 1855 | 0.011933 | -0.02829 |
| Kelpie_U | 1869 | -0.0494 | 0.015946 |
| Kelpie_U | 1881 | -0.01441 | -0.03563 |
| Kelpie_U | 1893 | -0.00495 | -0.00304 |
| Kelpie_U | 1951 | -0.00975 | -0.00795 |
| Kelpie_U | 1952 | 0.015839 | -0.00967 |
